# Regulatory Mechanisms Underlying Arsenic Uptake, Transport, and Detoxification in Rice

**DOI:** 10.3390/ijms241311031

**Published:** 2023-07-03

**Authors:** Anjing Geng, Wenli Lian, Xu Wang, Guang Chen

**Affiliations:** 1 Institute of Quality Standard and Monitoring Technology for Agro-Products of Guangdong Academy of Agricultural Sciences, Guangzhou 510640, Chinawangxuguangzhou@126.com (X.W.); 2 Key Laboratory of Testing and Evaluation for Agro-Product Safety and Quality, Ministry of Agriculture and Rural Affairs, Guangzhou 510640, China; 3 Guangdong Provincial Key Laboratory of Quality & Safety Risk Assessment for Agro-Products, Guangzhou 510640, China

**Keywords:** rice, arsenic, uptake, transport, detoxification, molecular mechanisms

## Abstract

Arsenic (As) is a metalloid environmental pollutant ubiquitous in nature that causes chronic and irreversible poisoning to humans through its bioaccumulation in the trophic chain. Rice, the staple food crop for 350 million people worldwide, accumulates As more easily compared to other cereal crops due to its growth characteristics. Therefore, an in-depth understanding of the molecular regulatory mechanisms underlying As uptake, transport, and detoxification in rice is of great significance to solving the issue of As bioaccumulation in rice, improving its quality and safety and protecting human health. This review summarizes recent studies on the molecular mechanisms of As toxicity, uptake, transport, redistribution, regulation, and detoxification in rice. It aims to provide novel insights and approaches for preventing and controlling As bioaccumulation in rice plants, especially reducing As accumulation in rice grains.

## 1. Introduction

Arsenic (As) is a metalloid pollutant commonly found in the natural environment. As early as 2004, it was listed as a class I carcinogen by the International Agency for Research on Cancer [1], and both natural phenomena (e.g., rock weathering, volcanic eruptions, and geothermal activity) and human activities (e.g., mining and smelting, pesticide production and application, landfill, and leather dye manufacturing) can lead to As pollution [2,3,4,5]. As pollution in the soil and irrigation water has been successively reported in many countries, such as India, Bangladesh, Vietnam, and China [6,7]. Among them, areas with relatively serious As pollution problems in the groundwater are South Asian countries, India and Bangladesh, and Southeast Asian countries, located around the Mekong Delta in Vietnam. More than 150 million people face the threat of As pollution in their drinking water [8,9]. With the rapid industrial and agricultural development, the area of soil and water bodies polluted by As is increasing yearly, and the degree of pollution is increasingly aggravated, which has aroused widespread concern worldwide [10]. 

Even if the As content is low, toxicological effects will still be generated after long-term exposure. According to statistics, more than ten million people worldwide are gradually being chronically poisoned by As, especially in South and Southeast Asian countries [11]. Currently, there are more than 200 arsenic compound types on Earth, including inorganic arsenic, methylated arsenic, arsenocholine, arsenosugars, arsenobetaine, and many other arsenic species in the environment. Arsenic-containing pesticides include lead arsenate, sodium arsenite, calcium arsenite, zinc arsenite, and arsenate [3], while As(III) (arsenite) is the major As species in the gold mining area [5]. The different As species have variable levels of toxicity. The order of toxicity of common arsenic compounds is: TMA(V) (trimethylarsine) < DMA(V) (dimethylarsinic acid) < MMA(V) (monomethyl-sonic acid) < As(V) (arsenate) < As(III) [12]. Arsenic in soil mostly exists in the form of As(V) or As(III). In flooded paddy fields, As(V) is easily reduced to As(III), which is more mobile [13]. Rice is the staple food of 350 million people in the world. Due to its growth characteristics, compared with other cereal crops such as wheat, barley, and corn, the plants and grains have a higher efficiency in the uptake of As from the environment [14]. The major As species in the grains is inorganic As [mainly As(III)]. High concentration of As results in toxicity, affecting the growth, development, and metabolic processes of rice, reducing plant resistance, biomass production, and yield [15,16]. In severe cases, the content of As in rice grains and rice products may exceed the maximum containment level [17], posing a serious threat to food security. It is specifically manifested as cytotoxicity to animals and humans [12], which in turn causes kidney, bladder, lung, and skin cancer, Bowen ‘s disease, hyperkeratosis, coronary heart disease, bronchiectasis, and other diseases [1,2].

**Figure 1:**
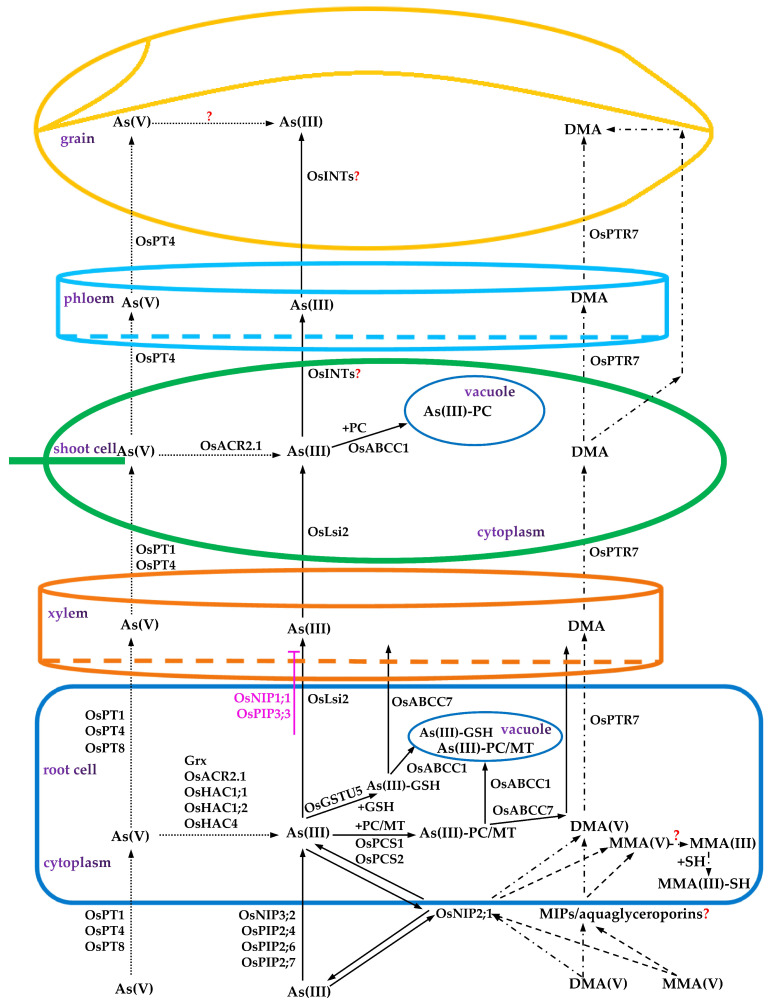
Schematic diagram for the uptake, transport, redistribution, and detoxification mechanisms of different arsenic (As) species in rice. (“—”, “…”, “﹎”, and “﹍” represent the influx, transport, efflux, and metabolic pathways of the As(III), As(V), DMA, and MMA species in different organs of rice, respectively. “→” indicates promoting effects. “⟂” indicates an inhibitory effect. “?” indicates possible but not fully clarified related physiological and biochemical processes or functional proteins involved).

Substantial progress has been made in research on the uptake and long-distance transport mechanisms of As in the soil for various crops [18]. This review comprehensively elaborates on As uptake, transport, and detoxification mechanisms in rice. The functional proteins involved in the uptake, transport, chelation, and vacuolar compartmentation of different As species in rice and their corresponding pathways are shown in Figure 1. This compilation and in-depth discussion and knowledge provide the theoretical support for formulating a solution to the problem of As pollution in the soil–rice system, cultivating rice with low As accumulation, and improving rice quality and safety.

## 2. The Toxic Effects of As

Arsenic toxicity affects the growth of rice roots and interferes with their normal physiological and metabolic functions, resulting in shorter roots, dwarf plants, and withered, curled, and necrotic leaves. Moreover, a number of growth parameters, such as leaf area, the number of leaves, the number of flowers, stem length, or root length, were observed to be reduced. Eventually, As stress leads to growth retardation and deterioration, and senescence of photosynthetic organs, and affects the transportation of water and mineral elements in vascular tissues [19,20]. Meanwhile, the uptake of nutrient elements in rice is reduced due to competitive inhibition, thereby decreasing biomass production and yield [20,21,22]. 

Arsenic toxicity leads to reactive oxygen species (ROS) accumulation, which in turn causes cell membrane lipid peroxidation, cell damage or death, damage to DNA and protein structure, and stomatal conductance reduction [23,24,25,26,27,28,29,30]. At the same time, reactive nitrogen species (RNS) are also induced by As toxicity, leading to nitrification stress and inhibition of respiration [31]. 

Arsenic inactivates enzymes by affecting their sulfhydryl groups or displacing essential ions at the dynamic binding site of the enzyme, hindering its catalytic function. Arsenic toxicity can directly or indirectly induce excessive ROS and RNS production, leading to severe oxidative damage, membrane leakage, increased malondialdehyde production, and inactivation of functional enzymes [24,25,29]. After rice plants are subjected to As treatment, their chlorophyll content, the photochemical quantum yield of photosystem II, and electron transfer rate are all decreased [32,33]. Thus, their photosynthesis was inhibited [34]. Photosynthetic pigments are damaged when As concentrations exceed 9 mg kg^−1^ [35]. As(III) binds to the sulfhydryl (-SH) and amino groups of proteins, modifying their structure and thereby affecting the relevant biochemical functions [36]. As(V), as a phosphate analog, affects the phosphate metabolism in rice [e.g., phosphate uptake, phosphorylation, and adenosine triphosphate (ATP) synthesis] and thereby inhibits photosynthesis and plant growth and development. The effects of As on the physiological and biochemical processes of rice are influenced by many factors, such as the As content, the As species, application or exposure method, exposure time, rice variety, uptake pathway, and accumulation capacity [28]. 

Arsenic enters into rice and produces toxic effects, affecting phenotype, physiological and biochemical metabolism, and yield. Therefore, revealing the molecular pathway of As uptake and transport in vivo will lay a foundation for the formulation of strategies to alleviate As toxicity in rice.

## 3. The Molecular Mechanisms of As Uptake, Transport, and Redistribution in Rice

The uptake of As from the soil by rice depends on its soil concentration, speciation, and other parameters in the rhizosphere. Under flooding conditions, due to the chemical reduction and dissolution of iron minerals, the most abundant As species in the paddy field soil solution is As(III) [37], usually accounting for 70% to 90%, and the rest is As(V), as well as small concentrations of methyl arsenic (including MMA and DMA). The order of different As species in which they are taken up by rice from the soil is As(III) > As(V) > DMA(V) > MMA(V) [38]. In view of the differences in chemical properties, the uptake mechanisms of various As species by rice roots are also different. Arsenic taken up by the root system is transported to the shoots and accumulated in the leaves, thereby affecting the rice plant’s tolerance to As. Similar to other mineral nutrients, a portion of the uptaken As is redistributed into rice through a transport system. The long-distance transport and redistribution of As are finely regulated. In recent years, several functional genes related to As uptake, transport, and redistribution have been identified and cloned (Table 1), laying the foundation for revealing the molecular regulation mechanism of As stress responses and tolerance in rice.

### 3.1. As Uptake

#### 3.1.1. Uptake of Arsenite in Rice

In flooded paddy fields with anaerobic conditions, the As(V) adsorbed on the iron plaque is reduced to As(III), which is more mobile and soluble, improving As availability. As(III) has a higher dissociation constant (pKa = 9.2), and it mainly exists in the form of undissociated neutral molecules (arsenous acid) at pH < 8 [60]. The rice root system takes up As(III) mainly through transmembrane transport conducted by certain major intrinsic protein (MIP) subfamily members, such as the nodulin26-like intrinsic proteins (NIPs), the plasma membrane intrinsic proteins (PIPs), and the tonoplast intrinsic proteins (TIPs) [49]. NIP channel proteins have lower As(III) selectivity and perform bidirectional transport. As a result, the influx and efflux of As(III) across the plasma membrane are mainly dependent on the intracellular As gradient [61]. Several aquaporins with As(III) transport activity have been reported, such as OsNIP1;1, OsNIP2;1, OsNIP2;2, OsNIP3;1, OsNIP3;2, OsNIP3;3, OsPIP2;4, OsPIP2;6, and OsPIP2;7 [44,48,62]. Among them, OsNIP2;1 (OsLsi1) and OsLsi2 play a key role in As(III) uptake and accumulation in rice [44]. OsLsi1 is a passive aquaporin that is permeable to various substrates, including silicon (Si) and As(III), and is the main constituent for As(III) uptake. The OsLsi1 protein is located on the distal side of the endodermis and exodermis membranes of the root system. At the same time, OsLsi2 is an aquaporin that mediates substrate efflux and is involved in As(III) translocation from rice root cells to the xylem [63,64]. The OsLsi2 protein is located on the proximal side of the endodermis and exodermis membranes [65]. This polar localization mode effectively ensures that As(III) crosses the endodermis and exodermis in roots and is eventually transported to the xylem, which results in xylem loading and transport to the shoot [63,65]. This mode of translocation is one important reason for the high Si and As(III) uptake capacities in rice [14,66]. In rice plants, the loss of function of OsLsi1 and OsLsi2 significantly reduces the uptake of As(III) and its transport to shoots. In fact, the main function of OsLsi1 and OsLsi2 is to take up and transport Si [63,65]. Since arsenous acid’s structure and physical and chemical properties are similar to silicic acid (H_4_SiO_4_), As(III) can enter the rice root system through the Si transporters. *OsNIP2;2* (*OsLsi6*) can transport As(III) when expressed in *Xenopus* oocytes, but the As concentration in roots and shoots of its mutants was not significantly different from that of wild-type (WT) when the mutants were exposed to 2 μM As(III) for seven days. This indicated that *OsNIP2;2* has a small contribution to As(III) uptake, and this may be attributed to its very low expression in rice roots [45]. *OsNIP1;1* and *OsNIP3;1*, which belong to Class I and Class II NIPs, respectively, do not transport silicic acid, and their expression levels are very low under As(III) stress [45]. OsNIP3;2 is involved in the uptake of As(III) by the lateral roots [48], and OsNIP3;3 exhibits transport activity under As(III) stress [62]. OsNIP1;1 and OsNIP3;3 are also involved in As(III) efflux [44]. When *OsPIP2;4*, *OsPIP2;6*, and *OsPIP2;7* are overexpressed in *Arabidopsis thaliana*, short-term As(III) exposure causes active As influx and efflux in the roots, promoting biomass accumulation and improving As(III) tolerance, indicating that PIPs are involved in the uptake of As(III) [49]. In addition, small basic intrinsic proteins (SIPs), uncategorized intrinsic proteins (XIPs), and TIPs regulate As(III) uptake in different modalities [54,67]. The expression patterns of As(III) uptake-related genes are significantly different across different rice varieties and growth stages. Therefore, knowledge of the gene expression patterns between varieties with high and low As uptake and transportation efficiency and the differences in the spatial and temporal distribution of As is very important for formulating As stress mitigation strategies in rice.

#### 3.1.2. Uptake of Arsenate in Rice

Aerobic soils are dominated by As(V), which is strongly adsorbed on mineral soil components such as iron (hydroxides) oxides [68]. Unlike As(III), As(V) has similar physicochemical properties to phosphate and enters the rice roots competitively through phosphate transporters located at the root cell membranes. The rice phosphate transporter gene family includes 13 genes, *OsPT1* to *OsPT13* [69], and As(V) enters rice cells mainly through OsPT1, OsPT4, and OsPT8 [39,40,42,70]. OsPT4 is localized to the plasma membrane. *OsPT4* knockout resulted in a reduction of the As(V) content in rice roots by 50–55% compared to WT [40], while the accumulation of As(V) increased in *OsPT4* overexpression lines [40]. The phosphate transporter OsPT8 has a high affinity for As(V). In *OsPT8* overexpression rice lines, the maximum uptake of As(V) increased by 3–5 times [41,42], while its uptake in *Ospt8* mutants decreased by 33–57%, and their tolerance to As(V) stress increased by 100 times [42]. 

As(V) has a feedback regulatory effect on the phosphate uptake pathway, and the presence of As(V) inhibits the expression of genes induced by phosphorus deficiency, such as *OsPT2*, *OsPT4,* and *OsPT8*, thereby reducing As(V) uptake [48]. Thus, it is possible to reduce As(V) uptake in roots and improve As(V) tolerance in rice by inhibiting the high-affinity phosphate transport system [39].

#### 3.1.3. Uptake of Other As Species in Rice

Previous studies have indicated that plants can methylate As to produce various organic arsenic species [71]. However, rice plants lack this ability, and their methylated arsenic is a result of microbial-mediated rhizosphere methylation [72]. In the soil, sulfate-reducing bacteria (SRB) play an important role in the production of DMA. Depletion of rhizosphere Fe(III)-reducing bacteria (FeRB) and SRB populations results in reduced As bioavailability and a lower concentration of methylarsenoids in the grains under oxidative conditions [73]. 

Compared to inorganic arsenic, the amount of organic arsenic taken up by the roots is significantly reduced, partly due to the lower abundance of organic arsenic in the soil. When inorganic arsenic was added to the nutrient solution, the methylated arsenic content in the rice plants grown in the solution was less than 3% of the total arsenic. Moreover, under sterile hydroponic conditions, no methylated arsenic was detected in rice [74]. The molecular mechanism of organic arsenic uptake by rice is not fully elucidated yet. In rice, it may be potentially mediated through aquaporins in the roots [47]. OsLsi1 is the first protein reported to be involved in the uptake and transport of MMA and DMA [47]. The uptake rates of MMA and DMA in the *Oslsi1* mutant were 80% and 49% lower, respectively, compared to WT, while the *OsLsi2* mutation resulted in no significant changes to the uptake of MMA and DMA [47]. In addition, glycerol transporters (aquaglyceroporins) at the plasma membrane were shown to promote DMA and MMA uptake by rice roots [75]. 

Compared with the two inorganic arsenic species—As(III) and As(V), the uptake rate of methylated arsenic by rice roots is lower [76] and decreases with the increase in the number of methyl groups [77]. This is because transporters have a low affinity to organic arsenic, and the increased hydrophobicity results in a reduced methylated arsenic uptake rate. The As(V) uptake coefficient by rice roots is 2.5- and 5-fold higher compared to MMA and DMA, respectively [78]. pH may affect the uptake of DMA and MMA through a balance shift between dissociation and protonation [79]. The MMA and DMA dissociation constants (pKa) are 4.19 and 6.14, respectively. The increase in pH promotes the dissociation of MMA and DMA and reduces their uptake by rice roots, indicating that MMA and DMA enter rice mainly in undissociated speciation [47]. 

Different As species are absorbed by rice roots through different transporters, and the long-distance transport of As determines its accumulation in the shoots and grains. Therefore, studying the molecular mechanism of As transport from roots to shoots, excavating key functional genes and regulating them reasonably are conducive to reducing As content in the shoots of rice.

### 3.2. Arsenic Transport

Arsenic is transported over long distances through the xylem and phloem. It is generally believed that the xylem is the transport route of inorganic arsenic from roots to shoots [80], but few studies have documented As transport via the phloem. The mobility of inorganic arsenic in non-hyperaccumulating plants is generally low [60]. The rice plants have a high As(III) mobility in the xylem [80]. Even so, in a 2–4 d short-term experiment, only 10% of the ^73^As(III) taken up by the root system was transported to the aboveground part, and only 3.3% of that percentage (i.e., 0.33% of the total uptake by the root system) was transported to the grains. When As(III) was supplied directly to the flag leaves, 2% to 3% of the ^73^As taken up by the leaves was transported to the grains within 2 days [80]. According to a xylem sap analysis, oxidized As is the dominant As species in the xylem [with As(V) accounting for 86% and DMA for 14%], while reduced As [with As(III) accounting for 71% and AsGlu3(tris-As-glutathione) for 29%] was mainly distributed in the vacuoles of cells near the xylem [81]. As(III) in the xylem vessels is transported to the shoot by OsLsi2 [82]. *OsLsi2* loss of function mutation significantly inhibits the transport of As(III) transport to the xylem and its accumulation in the shoot, resulting in a decreased As(III) content in the grains. This indicates that As transport to the xylem is a key step in controlling As accumulation in shoots. *OsNIP1;1* and *OsNIP3;3* transporters at the plasma membrane of rice root cells restrict As(III) entry into the xylem by removing it from the stele [44]. OsNRAMP1, a member of the natural resistance-associated macrophage protein (NRAMP) transporter family, promotes the loading of As(III) to the xylem [83], and overexpression of *OsNRAMP1* in *Arabidopsis* could enhance the plant tolerance to As(III) [54]. *OsGrx_C7* regulates root growth and inhibits the transport of As from the roots to the shoot by downregulating the expression of *OsNIP1;1*, *OsNIP3;1*, *OsLsi1*, and *OsLsi2*, significantly improving rice tolerance to As(III) and mitigating As(III) accumulation in the grains [83]. In *Arabidopsis*, NIP7;1 is involved in As long-distance transport, and *NIP7;1* loss of function mutations lead to a significant decrease in As content in the xylem and phloem [84]. AtINT2 and AtINT4, two inositol transporters, play an important role in As(III) influx into the phloem [85]. Identifying homologous genes of these *Arabidopsis* genes in rice is important in order to verify whether they have similar As transport functions.

Phosphate transporters efficiently deliver As from the roots to the shoots through xylem loading or long-distance transport. X-ray absorption spectroscopy imaging was used to visualize and verify As(V) transport from the roots to the shoots through the xylem [86]. As(V) is taken up by the cells of the shoot via phosphate transporters [82]. The As(V) content in the shoots of *Ospt1* knockout mutants was 60% lower compared to WT, while *OsPT1* overexpression promoted the shoot accumulation of As(V) [39]. OsPT4 regulates the transport of As(V) in rice, with the *Ospt4* mutant exhibiting significantly reduced content of inorganic arsenic in the grains [70]. OsPT8 significantly increases the transport of As(V) to the shoots [87]. As(V) stress decreases the expression levels of *OsPT8*, *OsPT4,* and *OsPHO1;2* in rice roots, while it increases the expression of *OsPCS1* and, as a result, the phytochelatin content, indicating that As(V) enhances the compartmentation of As in root cell vacuoles, limiting its transport to the shoots [23]. Constitutive expression of *OsMATE2,* the rice multidrug and toxic compound extrusion (MATE) family member in tobacco, reduced the translocation coefficient of As(V) from the roots to the shoots by 33.3–39.6% [58]. 

Compared with inorganic arsenic, methylated arsenic species (especially DMA) exhibit a significantly increased mobility in the xylem and phloem [88]. This might be because, under the cytoplasmic pH conditions, MMA(V) and DMA(V) are kept in a favorable dissociated state [89]. Compared with As(III), DMA has a better transport ability due to its poor interaction with sulfhydryl (SH) groups resulting from its particular molecular structure [90]. Moreover, due to the lack of DMA(V)-PC(phytochelatin) conjugate formation, DMA(V) is readily transported between roots and shoots [79]. Unlike the uptake efficiency, the amount of methylated arsenic species migrated from the root system to the shoot increased with the increase in the methyl group numbers [77]. When the rice root system was treated with DMA, the ratio of As concentration in the grains to that in the stems and leaves was more than 100-fold higher than inorganic arsenic treatment [91]. However, the molecular mechanism of methyl arsenic transport in rice is still unclear, and only a few reports exist. The peptide transporter OsPTR7 in rice is involved in the long-distance transport of DMA. The expression level of *OsPTR7* is higher in rice leaves, stem nodes, and roots at the flowering and grain-filling stages. On the other hand, at the seedling stage, the expression of *OsPTR7* was higher in the shoots compared to the roots [55]. Another study demonstrated that the MMA and DMA contents in the shoots and xylem sap of the *Oslsi1* mutants were lower than those of WT. In contrast, the MMA and DMA contents in the same organs of the *Oslsi2* mutant were not significantly different from those of WT [47], indicating that OsLsi1 rather than OsLsi2 may be involved in the transport of organic arsenic from roots to shoots.

At present, there are many studies on the transport of As(III) in rice, while the molecular mechanism of long-distance transport of other inorganic arsenic and organic arsenic is relatively scarce; in particular, the regulation mechanism of different As species redistribution to the grains is still unclear. An in-depth discussion of this process is conducive to adopting effective strategies to ensure the quality and safety of rice.

### 3.3. Arsenic Redistribution

Arsenic transport to rice grains is mainly carried out through the phloem [92]. The loading and unloading of As in phloem are directly related to the redistribution of As from the shoots to the grains. There are only a few studies on the transporters involved in this process. Around 90% of As(III) and 55% of DMA were found to be transported from the leaves to the grains [93]. It has also been reported that 100% of MMA(V), 89% of DMA(V), 54% of As(III), and 56% of As(V) were transported to the grains via the phloem [94]. The disparities between the above two studies can be attributed to the different experimental methods used. The distribution mechanisms of organic and inorganic arsenic in rice grains differ significantly. Inorganic arsenic is mainly enriched in the vascular bundles of the grain epidermis, while DMA is more likely to enter the endosperm [92,95]. In the phloem, organic arsenic is more mobile than inorganic arsenic [46]. MMA/DMA can be accumulated in rice grains in twice as high concentrations compared to As(III)/As(V) [95,96]. 

In *Arabidopsis*, the inositol transporters AtINT2 and AtINT4 play an important role in As(III) accumulation in the seeds [85]. Whether similar transporters in rice are responsible for the transport of As(III) to the grain remains to be further confirmed. The As concentration ratio in the first stem node to that in the grains of the *Oslsi2* mutant was significantly higher compared to the WT [64]. Synchrotron micro-X-ray fluorescence (μ-XRF) imaging demonstrated that As(III) is mainly restricted in the phloem, the meristem, and internodes, limiting the entry of As(III) into grains. OsPCS1 and OsABCC1 inhibit As transport to the grains by sequestration and compartmentalization in the vascular bundle cell vacuoles of the stem nodes [97]. OsABCC1 is localized at the tonoplast membrane of the phloem in rice internodes. It restricts the transport of As to the grains by promoting the transport of the As(Ⅲ)-PC conjugate to the vacuole. The total arsenic content in the *Osabcc1* knockout mutant stem nodes was reduced, while the As content in the grains was increased [53]. *OsMATE2* expression was up-regulated 6-fold in the seeds developed under As(V) stress and was positively correlated with As content in mature grains. In rice, endosperm-specific silencing of *OsMATE2* reduced the As content in the grains by 36.9–47.8% [58]. *OsPTR7* is highly expressed in rice roots, leaves, and stem nodes during the seed-filling stage [55] and is involved in DMA accumulation in the grains. *OsPTR7* knockout can reduce the long-distance transport of DMA to rice grains, but the molecular mechanism needs to be further studied.

The distribution of different As species in rice grains has been studied, but the signal pathway and molecular regulation mechanism of As transport to grains are still unclear. As a crop with high enrichment of arsenic, the transport and distribution of As in different organs are precisely regulated. Excavating upstream regulatory elements and improving the molecular network of As stress response in rice provide theoretical support for the cultivation of low-arsenic rice varieties.

## 4. Regulatory Mechanisms

The R2R3 MYB transcription factor OsARM1 is a negative regulator of As(III) transport [59]. OsARM1 binds to the MYB binding site of the *OsLsi1*, *OsLsi2,* and *OsLsi6* promoters, inhibits their expression, and regulates the uptake and transport of As(III). In addition, OsARM1 regulates As transport by directly binding to the promoter regions of the rice ABC transporter genes [59]. The MYB transcription factor OsPHR2 positively regulates the expression of *OsPT8*, increasing the uptake and transport of As(V) [41]. Transcriptome sequencing revealed that WRKYs are involved in rice responses to As stress [98]. OsWRKY28 regulates As(V) accumulation in rice seedlings, root architecture, and flower fertility by regulating the dynamic equilibrium of jasmonic acid (JA) or other plant hormones. OsWRKY28 loss-of-function results in a reduction of As and P contents in rice seedlings [56].

MicroRNAs (miRNAs) are involved, as upstream regulators, in the response of plants to As stress. miRNAs mainly affect the accumulation of arsenic by regulating transcription factor activities and the expression of stress-responsive genes to affect the corresponding gene networks. For example, *miR156j*, *miR164f*, and *miR1851* were downregulated in rice seedlings treated with As(III), while the expression levels of their target genes *OsTIP1;1*, *OsTIP1;2*, *OsTIP1*, and *OsPIP1;2* were increased [99]. miR399 is a conserved phosphate starvation-responsive miRNA, and it was upregulated in rice roots subjected to As(V) stress, indicating the potential regulation of certain aquaporins and As(V) transporters by miRNAs. After treatment with 25 μM As(III) and 50 μM As(V), differentially expressed miRNAs were identified in rice varieties with different As accumulation [100]. *miR528* plays a leading role in regulating rice tolerance to As(III) [101]. The *miR528* overexpression line was more sensitive to As(III) stress, while *miR528* knock outs exhibited improved As(III) tolerance [101]. 

Post-translational modifications (PTMs) play an important role in the rapid regulation of enzyme activity and transport processes. Ubiquitination, one of the most important PTMs that is highly conserved in eukaryotes, directly or indirectly regulates the key enzymes involved in As(III) uptake, efflux, chelation, and vacuolar sequestration [such as Arsenical Resistance3 (Acr3p)] and protects cells from As toxicity [102,103]. Thus, knockout mutants of genes related to ubiquitination and the ubiquitination-triggered proteosome/endocytosis pathway exhibit increased sensitivity to As [103,104]. Transcription factors that regulate key genes associated with As tolerance are, on many occasions, regulated by ubiquitination-dependent degradation. Moreover, As transporters regulate As(III) uptake through ubiquitination-triggered endocytosis [105]. *OsHIR1* encodes a RING-type E3 ubiquitin ligase in rice, which is induced under high As stress [105,106]. Overexpression of *OsHIR1* in *Arabidopsis* resulted in decreased As concentrations in roots and shoots and enhanced As tolerance. OsHIR1 was shown to interact with the vacuolar As(III) aquaporin OsTIP4;1, resulting in OsTIP4;1 degradation, limiting As accumulation in the roots and enhancing As tolerance. *OsNLA1* similarly encodes a RING-type E3 ubiquitin ligase, which is mainly localized at the plasma membrane and is involved in phosphate homeostasis maintenance in rice by mediating the degradation of OsPT2 and OsPT8 [107]. As(V) stress induces the expression of *OsNLA1*, its mutation leads to a significant increase in OsPT8 protein content, As hyperaccumulation, and As(V) hypersensitivity in rice [43]. 

Additional As uptake and transport regulatory pathways have also been discovered in rice. For example, OsPHF1 affects the uptake and transport of As(V) by regulating OsPHR2 or phosphorus transporters such as OsPT8 [41]. When *OsGrx_C7* and *OsGrx_C2.1* were overexpressed in *Arabidopsis*, the expression levels of *AtNIP1;1*, *AtNIP2;1*, and *AtNIP7;1* increased, the accumulation of As in seeds and shoots decreased, and, consequently, As tolerance was enhanced [51]. Under anaerobic conditions, the alcohol dehydrogenase (ADH) activity was reduced in the *Osadh2* mutant, resulting in a decrease in pH in the rice root cells, inhibiting the silicate transporters OsLsi1 and OsLsi2, and thereby reducing the accumulation of As(III) in the shoots and rice grains [57]. 

Rice regulates As stress response through transcription factors, miRNAs, and PTMs. In addition, rice has evolved other detoxification mechanisms to alleviate As toxicity.

## 5. Detoxification Mechanisms

**Figure 2:**
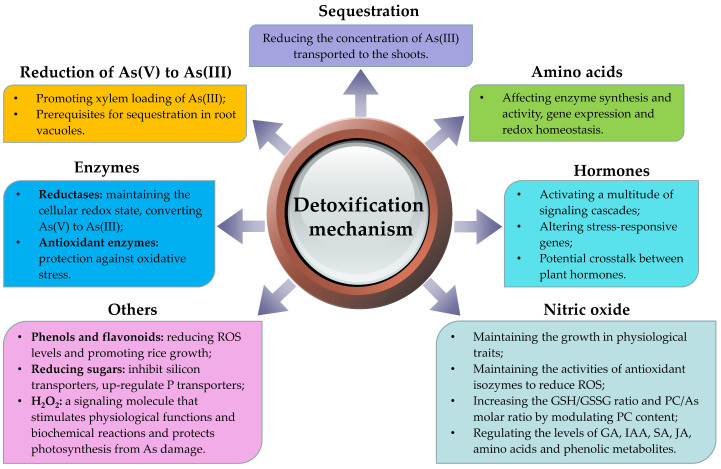
Arsenic detoxification mechanisms in rice.

As-detoxification processes in rice can be divided into two categories: external and internal. External detoxification mainly includes the application of iron, sulfur, and silicon, and immobilizing As in soil to reduce As absorption by rice roots [19,21]. Internal detoxification involves As species conversion and promotion of As efflux, conjugation, and vacuole compartmentalization through conjugation of As with metallothioneins (MTs), phytochelatins (PCs), cysteine-rich small-molecule polypeptides, and thiol-rich polypeptides [108,109]. In addition, under severe As stress, rice plants can activate defense systems (such as enzymes and non-enzymatic compounds) to repair ROS-induced damage [25,110,111]. Rice can also alleviate As toxicity by accumulating osmoregulatory compounds and their derivatives (such as organic acids, amino acids, and hormones) [110,112] (Figure 2). The reported functional genes related to As detoxification in rice are presented in Table 2.

### 5.1. As Speciation Conversion

The first step of As detoxification in rice is the reduction of As(V). Arsenate reductase catalyzes the reduction of As(V) to As(III). In rice, As(V) reduction is mainly carried out by OsHAC1;1, OsHAC1;2, OsHAC4, OsACR2.1, OsGrx, etc. [113,114,115,116], among which a key role is played by the As(V) reductase OsHAC1;1, located in the epidermis, root hairs, and pericycle cells, and OsHAC1;2, located in the epidermis and endodermis/exodermis cells [113]. OsACR2.1 has a dual function as a phosphatase and arsenate reductase, and its expression level was significantly increased in rice roots and shoots treated with As(V) [116]. After As(V) is reduced, As(III) can be released back to the external environment through the efflux system. Overexpression of *OsHAC1;1* or *OsHAC1;2* promotes As(V) reduction and the subsequent As(III) efflux and reduces As accumulation in the shoots. In contrast, in knock-out mutants, these genes exhibit the opposite phenotypes. Field experiments have shown that the As content in the grains of *OsHAC1;1* and *OsHAC1;2* overexpression lines were 20% lower than that of WT [113]. Mutation of *OsHAC4* led to a decrease in As(V) reduction and As(III) efflux in rice roots and significantly increased As accumulation in shoots. At the same time, overexpression of *OsHAC4* reduced As accumulation in vivo and improved rice As(V) tolerance [115]. Therefore, the reduction of As(V) to As(III) in rice roots and the subsequent As(III) efflux or the formation of As(III)-thiol conjugates are important mechanisms for As(V) detoxification, and they are also important factors affecting the total As(III) content in rice grains.

### 5.2. Chelation and Vacuolar Compartmentalization

Sulfhydryl (-SH)-rich compounds [such as glutathione (GSH) and PCs] and MTs can easily chelate As(III) that enters the root system, forming As(III) complexes, which are then compartmentalized to vacuoles via vacuole membrane-localized transporters. Such compounds play critical roles in enhancing rice tolerance to As stress [108]. Therefore, the genes involved in the regulation of GSH, PC, and MT synthesis pathways can directly or indirectly affect As accumulation and tolerance in rice. For example, the phytochelatin synthases OsPCS1 and OsPCS2 in rice catalyze PC biosynthesis, thereby reducing As accumulation and alleviating As toxicity [109,117,118,119]. OsGSTU5 is involved in the chelation of As(III) with GSH, which results in the compartmentalization of the conjugates into root cell vacuoles [121]. 

As(III) easily binds to proteins or polypeptides containing two or three adjacent sulfhydryl groups. There are many As(III)-PC or As(III)-PC/GSH conjugates formed in rice. The *OsABCC1* gene encodes a key transporter for the vacuolar sequestration of As(III)-PC conjugates. The *Osabcc1* mutant is more sensitive to As, and its As content in the grains is 13–18 times higher compared to WT [52]. OsABCC7 also possesses As(III)-GSH and As(III)-PC transport activities, while As stress inhibits its expression. Knockout of *OsABCC7* can significantly reduce the As concentration in the xylem sap and shoots [53]. OsCLT1 is involved in GSH homeostasis maintenance by mediating the export of γ-glutamylcysteine and GSH from the plastid to the cytoplasm, thereby affecting the detoxification of As in rice [120]. *OsCLT1* loss of function leads to a reduction in GSH and PC in the cytoplasm and inhibits the reduction of As(V). Moreover, the accumulation of As in the shoots of the *Osclt1* mutant is significantly higher compared to WT [120]. Mutation of *OsOASTL-A1* reduces the GSH and PC content, and the mutant’s seedlings are more sensitive to As [125]. Overexpression of *OsSultr1;1* in *Arabidopsis* can promote GSH production, increase the activity of the antioxidant system, promote the biosynthesis of PCs, and facilitate the chelation of As and its compartmentalization into the vacuoles [122]. Expression of the phytochelatin synthase gene *CdPCS1* from the aquatic As-accumulator plant *Ceratophyllum demersum* in rice increased the synthesis of PCs and the accumulation of As in roots and shoots, while the As content in the grains and hulls was significantly reduced [126]. 

### 5.3. Enzymes

The enzymes involved in As detoxification in rice can be largely divided into reductases and antioxidant-metabolism enzymes. Reductases mainly include glutaredoxin (GRX), GSH, and glutathione reductase (GR). On the one hand, these reductases can maintain the Redox homeostasis in the plant; on the other hand, they can promote the reduction of As(V) to As(III), which then is sequestered to the vacuoles to relieve As toxicity in the cytoplasm. Antioxidant enzymes mainly include superoxide dismutase (SOD), catalase (CAT), GPX, GR, and ascorbate peroxidase (APX) [110,127,128], mainly involved in the removal of ROS. Rice plants can reduce the oxidative damage of free radicals by increasing the activity of antioxidant enzymes [127,129]. Moreover, As(V) can upregulate genes encoding antioxidant enzymes [130]. 

Under As stress, the expression of four glutaredoxin and two glutathione-S-transferase (GST) encoding genes in As-sensitive rice varieties were higher compared to the resistant rice varieties [111]. GRX, which has As(V) reductase activity, can maintain GSH content and assist As(III) efflux [131]. The GRX gene *Os01g26912* was found to be specifically expressed in the shoots of rice treated with As(III), indicating the presence of As(III)- and As(V)-specific gene responses in rice [130]. OsWNK9 may regulate As(III)-induced oxidative stress by activating the antioxidant system. Its overexpression in *Arabidopsis* could enhance the activity of antioxidant enzymes, reduce ROS accumulation, and improve tolerance to As(III) [123]. In addition, antioxidant enzymes such as APX, dehydroascorbate reductase (DAR), monodehydroascorbate reductase (MDAR), and GR involved in the ascorbic acid–glutathione cycle play an important role in protecting rice cells from oxidative damage caused by As toxicity [132]. 

### 5.4. Amino Acids

Under As stress, the rice plant synthesizes metabolites, such as proline, histidine, cysteine, and certain amino acids [110], which affect several As detoxification aspects, such as the synthesis and activity of As detoxification-associated enzymes, gene expression, and redox homeostasis. Proline contributes to protein, DNA, and cell membrane structure stability and can reduce ROS-mediated oxidative damage [112]. Proline accumulation in rice seedlings is tightly correlated to the concentrations of As(III) and As(V) applied in treated plants [133]. In addition, proline promotes the synthesis of PCs and the vacuolar compartmentation of As, improving the tolerance of rice plants to As [134]. The proline content in *OsWNK9* overexpression lines under As(III) stress was significantly higher than in WT, and their biomass increased by 52–58% [123]. Additionally, L-glutamic acid, as a signaling molecule, can increase nitrogen assimilation-related enzyme activity in rice plants under As stress and enhance antioxidant and proline metabolism, thereby alleviating As toxicity [135,136]. 

### 5.5. Nitric Oxide (NO)

NO relieves As stress by increasing antioxidant enzyme activities, promoting the biosynthesis of PCs and sulfate uptake in roots, enhancing the synthesis of amino acids and sulfhydryl compounds, and maintaining micronutrient and other trace element homeostasis. NO regulates the activity of antioxidant enzymes through post-translational modifications and reduces As-induced oxidative damage [137]. NO can affect the levels of plant growth regulators, such as gibberellic acid (GA), auxin (IAA), salicylic acid (SA), and JA, amino acids, and phenolic metabolites, thereby reducing As accumulation [138,139,140]. Under As(III) stress, NO regulates the expression of rice genes encoding metal transporters (*OsNAS3*, *OsZIP7*, and *OsVIT1*) and genes involved in hormone metabolism (*OsPIN9*, *OsIPT3*, *OsGA2ox4*, *OsNCED2*, *OPR1*, and *OPR2*) and secondary metabolism (*OsDHFR*, *CAD8*, and *OsLAC3*) [141]. NO promotes the expression of genes encoding PIN transporters and changes root architecture, thereby increasing the tolerance to As [141,142]. NO can also trigger the release of intracellular Ca^2+^, activate antioxidant enzymes, and thus reduce As-induced oxidative stress [143]. Nevertheless, the molecular mechanisms underlying the roles of NO in regulating antioxidant enzyme activities, the PC/As ratio, amino acid metabolism, etc., in rice are still not fully elucidated.

### 5.6. Hormones

Under As(III) stress, methyl jasmonate (Me-JA) can promote root growth, increase photosynthetic rate, reduce membrane damage, and regulate the expression levels of numerous genes related to As uptake, transport, and detoxification, such as *OsLsi1*, *OsLsi2*, *OsLsi6*, *OsNIP1;1*, *OsNIP3;1*, *OsINT5*, *OsNRAMP1*, *OsPCS2*, and *OsABCC2*, thereby alleviating As(Ⅲ) toxicity in rice plants [144]. SA is involved in abiotic stress signal transduction, as well as rice responses to As, effectively alleviating the oxidative damage and growth inhibition caused by As toxicity [145]. Under As stress, exogenous SA application promoted NO synthesis [146] and reduced As accumulation in shoots [147]. Exogenous application of SA and NO downregulated *OsLsi2* expression and reduced the transport of As to rice shoots [146,147]. SA interacts with ethylene and NO to promote rice photosynthesis and growth under As stress, and regulates the antioxidant defense system, improving As tolerance [148]. Furthermore, the expression levels of phosphorus transporter genes (*OsPT1*, *OsPT2*, *OsPT4*, and *OsPT8*) in strigolactone (SL) deletion mutants under As(V) stress were significantly higher compared to WT. In contrast, the GSH content, *OsPCS1* and *OsABCC1* expression levels, and SOD, APX, and CAT activities were significantly lower compared to WT, indicating that SLs are involved in alleviating As stress by regulating As(V) uptake, vacuolar compartmentation, GSH biosynthesis, and antioxidant defense responses in rice roots [149]. 

### 5.7. Other Small Molecules

In rice, metabolites whose levels are altered under As stress include phenolic compounds and flavonoids with antioxidant functions. The contents of metabolites such as 3-hydroxybenzoic acid, apigenin, and genistein increase while 2-hydroxycinnamic acid and trans-cinnamic acid decrease. Increasing root lignin content under As stress is another mechanism for reducing As accumulation. Overexpression of the rice type III peroxidase gene *OsPRX38* in *Arabidopsis* activates the RedOx signaling network and promotes lignin biosynthesis. An increase in root lignification hinders As uptake through apoplast lignification and reduces As accumulation [124]. Hydrogen peroxide (H_2_O_2_) accumulation induces the expression of SOD and APX-encoding genes, reduces the As-induced oxidative stress, and promotes the expression of the *psbA* and *psbB* genes, thereby enhancing photosynthesis, and thus improving the tolerance to As stress [150]. Moreover, an increase has been reported in the concentration of reducing sugars in As-stressed rice seedlings [151], as well as the contents of total sugars, reducing sugars, and other carbohydrates in the grains [152]. Therefore, enhancing the accumulation of soluble sugars can alleviate As toxicity.

## 6. Conclusions and Prospects

Arsenic pollution and accumulation in rice are tightly linked to food security in the world and human health. Limiting As uptake or redistributing it to grains effectively reduces As content in rice and reveals the molecular regulatory mechanisms underlying As uptake, transport, and redistribution, laying the foundation for further manipulation of these processes to achieve a higher reduction in As accumulation and toxicity in rice. Although significant progress has been realized in this field, only a few key functional genes have been identified and cloned, and the genetic regulatory networks are not well characterized. 

In terms of As uptake, reducing As influx into rice is one of the key approaches to reducing As accumulation. Fertilization, the application of exogenous regulatory compounds, and management of the growth environment are effective measures to prevent As uptake. Therefore, studying the regulatory mechanisms of existing regulatory compounds and finding new regulatory compounds is important for further reducing As uptake. At the same time, As uptake in rice is affected by various factors, both internal and external (such as the rice variety, soil pH, and the iron and sulfate concentration in the soil). Therefore, instead of focusing on the analysis of single factors, it is necessary to explore the mechanism for multiple factors influencing As uptake to lay the theoretical foundation for future applied management practices. In addition, the uptake rate of methylated As in rice is low, and the production of methylated arsenic and volatile As is linked to the presence and activities of soil microorganisms. Screening of microorganisms that are efficient in arsenic methylation and their practical application in production are also important endeavors to reduce As uptake in rice. Some transporters for As uptake have been identified so far. Are there other uptake-related genes? The research on the efflux of As(Ⅲ) by OsLsi2 is relatively comprehensive. Are there other efflux transporters of As(Ⅲ) or other As species in rice? These transporters potentially significantly affect the content of As in root cells, and follow-up research can focus on this aspect. 

In terms of As transport and redistribution, current research focuses on the long-distance transport of As(Ⅲ), while the molecular mechanisms and related metabolism underlying the transport of other As species between various tissues and organs of rice need to be further investigated. How are various As species transported into the seed coat, aleurone layer, endosperm, and embryo? How can we control the concentration and proportion of As in each seed tissue type? Is there an As speciation transformation in the kernel? Can the various As species combine with starch and protein in the grains? Answering these research questions can provide valuable information for the fine processing of rice grains. 

In terms of As accumulation regulation, there are currently few studies on the transcriptional and post-translational regulatory mechanisms of transporters involved in As uptake, transport, and redistribution. Discovering new upstream regulatory factors and studying their mechanisms of action will help deepen our understanding of the molecular signaling pathways involved in rice responses to As stress. 

In terms of As detoxification, (1) there are many reports on the reduction of As(Ⅴ) to As(Ⅲ) and the chelation of As(Ⅲ) and vacuole compartmentalization to alleviate the toxicity of As in rice. Is the As compartmentalized in the vacuole appropriately contained, or can it leak from the vacuole? If this occurs, is it exuded and transported in the form of arsenic monomer or arsenic chelate? Are there other As species sequestered by PCs and MT into the vacuole? Are there further phase transitions of As transported into the vacuole? (2) Various enzymes or metabolites in rice positively affect As detoxification. The feedback mechanisms and influencing factors of each metabolite on As-associated metabolic pathways need to be further studied. (3) Can various factors, such as nutritional and environmental factors that affect rice growth, that mitigate As toxicity be explained through large-scale biology analyses, model simulations, and principal component analyses? (4) The uptake, transport, and metabolism of As vary in the different growth stages of rice. The dynamic analysis of As throughout the growth period is of great significance in carrying out effective prevention and control measures in each growth stage. With our continuous efforts to understand the genetic and molecular mechanisms of rice responses to As stress, more downstream functional genes and upstream regulatory elements of As uptake and transport will be identified. By assessing and exploiting elite allelic variants in rice germplasm, new rice varieties with low As accumulation could be developed using gene-editing technologies.

## Figures and Tables

**Table 1:** Genes related to As uptake, transport, and redistribution in rice.

Gene Name	Gene ID	Main Expression Tissues or Organs	Subcellular Localization	Function	Ref.
*OsPht1;1* (*OsPT1*)	LOC_Os03g05620	Root, shoot	Plasma membrane	Involved in As(V) uptake from the soil or apoplast and involved in root-to-shoot As(V) transport	[39]
*OsPht1;4* (*OsPT4*)	LOC_Os09g37200	Root, shoot	Plasma membrane	Takes up and transports As(V) from the root to the shoot	[40]
*OsPht1;8* (*OsPT8*)	LOC_Os10g30790	Root, shoot	Plasma membrane	Transports As(V) into the root or xylem	[41,42]
*OsPHF1*	LOC_Os07g09000	Root	Plasma membrane	Regulates OsPT8 for the uptake and transport of As(V)	[41]
*OsPHR2*	LOC_Os07g25710	Root, shoot	Plasma membrane	Regulates phosphate transporters to affect As(V) transport to the root and xylem	[41]
*OsNLA1*	LOC_Os07g47590	Roots, shoot	Plasma membrane	Regulates As(V) uptake and tolerance mainly via regulating the amount of phosphate transporters	[43]
*OsNIP1;1*	LOC_Os02g13870	Root	Plasma membrane	Restricts As(III) loading into the xylem by providing a route for As exudation from the stele	[44]
*OsNIP2;1* (*OsLsi1*)	LOC_Os02g51110	Root, leaf, anther	Plasma membrane	Translocates organic species of As into the root, shoot, xylem/translocates As(III) into the root	[45,46,47]
*OsLsi2*	LOC_Os03g01700	Root	Plasma membrane	Involved in As(III) transport out of the root cells toward the stele	[45]
*OsNIP2;2* (*Os Lsi6*)	LOC_Os06g12310	Root, leaf, shoot	Plasma membrane	Weak capacity to absorb As(III) in rice	[45]
*OsNIP3;1*	LOC_Os10g36924	Root, leaf, shoot, anther	Membrane	Weak capacity to absorb As(III) in rice	[45]
*OsNIP3;2*	LOC_Os08g05590	Leaf, root, anther	Plasma membrane	Transports As(III) into the root	[48]
*OsNIP3;3*	LOC_Os08g05600	Root	Plasma membrane	Restricts As(III) loading into the xylem by providing a route for As exudation from the stele	[44]
*OsPIP2;4*	LOC_Os07g26630	Root	Plasma membrane	Plays a role in the permeability of As(III) in *Arabidopsis*	[49]
*OsPIP2;6*	LOC_Os04g16450	Root, leaf, shoot, anther	Plasma membrane	Transports As(III) into oocytes, and effluxes As(III) from the root of *Arabidopsis*	[49]
*OsPIP2;7*	LOC_Os09g36930	Root, leaf, anther	Plasma membrane	Plays a role in the permeability of As(III) in *Arabidopsis*	[49]
*OsGrx_C7*	LOC_Os01g27140	Root, shoot	/	Alters expression of As(III) transporters (aquaporins) in rice and *Arabidopsis*	[50,51]
*OsABCC1*	LOC_Os04g52900	Root, shoot, grain, leaf, nodes, peduncle, rachis	Tonoplast	Limits As transport to the grains by sequestering As in the vacuoles	[52]
*OsABCC7*	LOC_Os04g49900	Xylem parenchyma cells in the stele region of the primary and lateral roots	Plasma membrane	Involved in the translocation of As from the roots to the shoots in rice, likely by mediating the efflux of As(III)-thiol complexes for xylem loading	[53]
*OsNRAMP1*	LOC_Os07g15460	Root	Plasma membrane	Assists As(III) xylem loading for root-to-shoot mobilization in *Arabidopsis*	[54]
*OsPTR7*	LOC_Os01g04950	Root, shoot, leaf, nodes	Plasma membrane	Transports DMA to xylem, phloem, and grain	[55]
*OsWRKY28*	LOC_Os06g44010	Strong expression in the root tip, lateral root, and reproductive organs	Nuclei	Regulates As(V) accumulation in the shoot	[56]
*OsADH2*	LOC_Os11g10510	Root	/	Regulates silicate transporters to influence As(III) contents in aerial tissues of rice	[57]
*OsMATE2*	LOC_Os05g48040	Root, leaf, seed	/	Modulates As accumulation in rice grain	[58]
*OsARM1*	LOC_Os05g37060	Phloem of vascular bundles in basal and upper nodes	Nucleus	Regulates As(III) uptake and root-to-shoot translocation	[59]

(“/” indicates not yet reported).

**Table 2:** Arsenic detoxification-related genes in rice.

Gene Name	Gene ID	Main Expression Tissues or Organs	Subcellular Localization	Function	Ref.
*OsHAC1;1*	LOC_Os02g01220	Root, shoot	Cytoplasm, nucleus	Reduces As(V) to As(III)	[113,114]
*OsHAC1;2*	LOC_Os04g17660	Root	Cytoplasm, nucleus	Reduces As(V) to As(III)	[113]
*OsHAC4*	LOC_Os02g06290	Root	Cytoplasm, nucleus	Reduces As(V) to As(III)	[115]
*OsACR2.1*	LOC_Os10g39860	Root, shoot	Cytoplasm, nucleus	Reduces As(V) to As(III)	[116]
*OsPCS1*	LOC_Os05g34290	Root, shoot, leaf, grain	Cytosol	Catalyzes the formation of phytochelatins that form As(III)-phytochelatin conjugates	[117,118]
*OsPCS2*	LOC_Os06g01260	Root, shoot, leaf, grain	Cytosol	Catalyzes the formation of phytochelatins that form As(III)-phytochelatin conjugates	[117,118,119]
*OsGrx_C2.1*	LOC_Os02g40500	Root, shoot	/	Involved in redox regulation and protection under oxidative stress/alters the transcripts of *AtNIPs* in *Arabidopsis*	[50]
*OsCLT1*	LOC_Os01g72570	Root	Envelope membrane of plastids	Maintains glutathione (GSH) homeostasis probably by mediating the export of γ-glutamylcysteine and GSH from the plastids to the cytoplasm, thereby affecting As detoxification in rice	[120]
*OsGSTU5*	LOC_Os09g20220	Root	Cytoplasm	Chelates As with GSH and sequesters it into the root cells vacuoles	[121]
*OsSultr1;1*	LOC_Os03g09970	/	/	Maintains ROS homeostasis, promotes the chelation of As with GSH and its sequestration into the root-cell vacuoles, and limits the acropetal translocation of As towards the shoot	[122]
*OsWNK9*	LOC_Os12g06490	Plant tissues	Nucleus	Involved in the regulation of arsenite-induced oxidative stress management by activating the antioxidant system and osmotic adjustment processes	[123]
*OsPRX38*	LOC_Os03g13210	Root	Apoplast	Enhances As(III) and As(V) tolerance by increasing ROS detoxification/reduces As accumulation due to high lignification	[124]
*OsOASTL-A1*	LOC_Os03g53650	Root	Cytosol	Plays an important role in non-protein thiol biosynthesis in roots for As detoxification	[125]

(“/” indicates not yet reported).

## Data Availability

Not applicable.

## Abbreviations

ABC	ATP-binding cassette
Acr3p	arsenical resistance3
APX	ascorbate peroxidase
As	arsenic
As(III)	arsenite
As(III)-GSH	As(III) glutathione conjugate
As(III)-PC/MT	As(III)-phytochelatin/metallothionein conjugates
As(V)	arsenate
AsGlu3	tris-As-glutathione
ATP	adenosine triphosphate
CAD8	cadherin-8
CAT	catalase
DAR	dehydroascorbate reductase
DMA	dimethyl arsenic acid
DNA	deoxyribonucleic acid
FeRB	Fe(III)-reducing bacteria
GA	gibberellin
GR	glutathione reductase
Grx	glutaredoxin
GSH	glutathione
H_2_O_2_	hydrogen peroxide
IAA	auxin
INTs	inositol transporters
JA	jasmonate
MATE	multidrug and toxic compound extrusion
Me-JA	methyl jasmonate
MIPs	membrane intrinsic proteins
miRNAs	micro ribonucleic acids
MMA(III)-SH	monomethyl arsenic acid sulfhydryl conjugate
MMA	monomethyl arsenic acid
MT	metallothionein
NIPs	nodulin 26-like intrinsic proteins
NO	nitric oxide
NRAMP	natural resistance-associated macrophage protein
OPR	oxophytodienoate reductase
OsABCC	C-TYPE ABC subfamily transporter
OsACR2.1	arsenate reductase 2.1
OsADH2	alcohol dehydrogenase 2
OsARM1	arsenite-responsive MYB1
OsCLT1	CRT-like transporter 1
OsDHFR	dihydrofolate reductase
OsGA2ox4	GA 2-oxidases 4
OsGSTU5	glutathione-S-transferase 5
OsHAC	high arsenic content
OsHIR1	heavy metal-induced ring E3 ligase 1
OsIPT3	adenylate isopentenyl transferase 3
OsLAC3	laccase gene 3
OsLsi	low silicon
OsNAS3	nicotianamine synthase gene 3
OsNCED2	9-cis-epoxycarotenoid dioxygenase 2
OsNLA1	nitrogen limitation adaptation 1
OsOASTL-A1	O-acetyl serine(thiol) lyase 1
OsPCS	phytochelatin synthase
OsPHF1	phosphate transporter traffic facilitator 1
OsPHR2	phosphate starvation response 2
OsPIN9	probable auxin efflux carrier component 9
OsPRX38	peroxidase 38
OsPT(OsPht)	phosphate transporter
OsPTR7	putative peptide transporter 7
OsWNK9	with no lysine 9
OsVIT1	vacuolar membrane transporter 1
OsZIP7	ZRT and IRT-like protein 7
PCs	phytochelatins
PIPs	plasma membrane intrinsic proteins
PTMs	post-translational modifications
psb	photosystem subunit
RING	really interesting new gene
RNS	reactive nitrogen species
ROS	reactive oxygen species
SA	salicylic acid
SH	sulfhydryl
Si	silicon
SIPs	small basic intrinsic proteins
SL	strigolactone
SOD	superoxide dismutase
SRB	sulfate-reducing bacteria
TMA(V)	trimethylarsine
TIPs	tonoplast intrinsic proteins
μ-XRF	synchrotron micro-X-ray fluorescence
WT	wild type
